# Updating Animal Welfare Thinking: Moving beyond the “Five Freedoms” towards “A Life Worth Living”

**DOI:** 10.3390/ani6030021

**Published:** 2016-03-14

**Authors:** David J. Mellor

**Affiliations:** Animal Welfare Science and Bioethics Centre, Institute of Veterinary, Animal and Biomedical Sciences, Massey University PN 452, Palmerston North 4442, New Zealand; d.j.mellor@massey.ac.nz; Tel.: +64-6-356-9099 (ext. 84024)

**Keywords:** animal welfare management, barren environments, enrichment, five domains, five freedoms, lives worth living, negative experiences, positive experiences, quality of life

## Abstract

**Simple Summary:**

The Five Freedoms were formulated in the early 1990s and are now well recognised as highly influential in the animal welfare arena. However, a marked increase in scientific understanding over the last two decades now shows that the Five Freedoms do not capture, either in the specifics or the generality of their expression, the breadth and depth of current knowledge of the biological processes that are germane to understanding animal welfare and to guiding its management. For example, this paper refers to some negative experiences that can never be eliminated, merely temporarily neutralised, because they are essential for eliciting behaviours upon which the survival of the animal depends. In addition, it refers to other negative experiences that relate to an animal’s responses to living in poor environments which require improvement, and also to how such experiences may be replaced by positive ones when particular improvements are introduced. For animals to have “lives worth living” it is necessary, overall, to minimise their negative experiences and at the same time to provide the animals with opportunities to have positive experiences. These observations have implications for reviewing and potentially updating minimum standards in codes of welfare. The paper ends with an up-to-date characterisation of the principal features of animal welfare, expressed largely in non-technical terms.

**Abstract:**

The Five Freedoms have had major impact on animal welfare thinking internationally. However, despite clear initial statements that the words ‘freedom from’ should indicate ‘as free as possible from’, the Freedoms have come to be represented as absolute or fundamental freedoms, even rights, by some animal advocate and other groups. Moreover, a marked increase in scientific understanding over the last two decades shows that the Freedoms do not capture the more nuanced knowledge of the biological processes that is germane to understanding animal welfare and which is now available to guide its management. For example, the named negative experiences of thirst, hunger, discomfort and pain, and others identified subsequently, including breathlessness, nausea, dizziness, debility, weakness and sickness, can never be eliminated, merely temporarily neutralised. Each one is a genetically embedded element that motivates animals to behave in particular ways to obtain specific life-sustaining resources, avoid or reduce physical harm or facilitate recovery from infection or injury. Their undoubted negativity creates a necessary sense of urgency to respond, without which animals would not survive. Also, the temporary neutralisation of these survival-critical affects does not in and of itself generate positive experience. This questions the commonly held assumption that good animal welfare will result when these internally generated negative affects are minimised. Animals may also experience other negative affects that include anxiety, fear, panic, frustration, anger, helplessness, loneliness, boredom and depression. These situation-related affects reflect animals’ perceptions of their external circumstances. Although they are elicited by threatening, cramped, barren and/or isolated conditions, they can often be replaced by positive affects when animals are kept with congenial others in spacious, stimulus-rich and safe environments which provide opportunities for them to engage in behaviours they find rewarding. These behaviours may include environment-focused exploration and food acquisition activities as well as animal-to-animal interactive activities, all of which can generate various forms of comfort, pleasure, interest, confidence and a sense of control. Animal welfare management should aim to reduce the intensity of survival-critical negative affects to tolerable levels that nevertheless still elicit the required behaviours, and should also provide opportunities for animals to behave in ways they find rewarding, noting that poor management of survival-critical affects reduces animals’ motivation to utilize such rewarding opportunities. This biologically more accurate understanding provides support for reviewing the adequacy of provisions in current codes of welfare or practice in order to ensure that animals are given greater opportunities to experience positive welfare states. The purpose is to help animals to have lives worth living, which is not possible when the predominant focus of such codes is on survival-critical measures. Finally, an updated characterisation of animal welfare that incorporates this more accurate understanding is presented.

## 1. Introduction

The Five Freedoms (Table 1) are very well known internationally. They have often been and still are referenced, for example, when the principal features of animal welfare are being outlined in policy statements (e.g., [1,2,3]), in authoritative treatises (e.g., [4,5,6]), and when described for lay audiences by animal welfare NGOs (e.g., [7,8,9,10]). They have certainly influenced animal welfare legislation, such as the UK Animal Welfare Act 2006 [3], as well as many accreditation or assurance schemes for farm animal welfare (e.g., [8,11,12]). This was in part because, when formulated, they were the first to detail the broader dimensions of animal welfare by incorporating subjective experiences, health status and behavior [13]. More specifically, they referred to thirst, hunger, fear, distress, discomfort, pain, malnutrition, injury, disease and behavioural expression (Table 1). They also usefully highlighted animal management actions, known as the Five Provisions (Table 1), which were then included in codes of practice designed to improve animal welfare [12,14]. Many early and current codes still show clear evidence of this (e.g., [15,16,17,18]).

The key features of the Five Freedoms paradigm (Table 1) have remained virtually unaltered since 1993/1994 when John Webster formulated it [12,14] by extending the first version which focused mainly on the space that should be made available to animals as emphasised by the earlier Brambell Committee Report [19]. This broader Freedoms paradigm was not intended to represent ideal or unattainable states, nor an absolute standard for compliance with acceptable principles of good welfare [13]; rather, it was to be a checklist by which to assess the strengths and weaknesses of husbandry systems [12]. With regard to the first four Freedoms, the phrase “freedom from” was intended in each case to indicate that animals should be kept “as free as possible from” the negative states identified, as it was clearly understood that complete freedom from these states during the life of an animal was not achievable [12].

The Five Provisions aligned with the Freedoms (Table 1) have rarely been named as such, as the Five Freedoms has been the dominant term in use. The Provisions, which it might be argued have been more influential than the Freedoms, were aimed at practical measures for securing the Freedoms understood within the limits of the caveats noted above.

In 2012, the Farm Animal Welfare Council (FAWC) stated on its website that the Five Freedoms “define ideal states rather than standards for acceptable welfare” … and together with the aligned Provisions … “form a logical and comprehensive framework for analysis of welfare within any system together with the steps and compromises necessary to safeguard and improve welfare within the proper constraints of an effective livestock industry” [7]. This statement reflected the then longstanding acceptance by informed members of the farming sector that the Five Freedoms paradigm could be usefully applied to guiding the welfare management of their livestock (e.g., [8,15,16,18,20]). Furthermore, animal welfare NGOs, which usually focus on a wider range of animal sectors, also adopted the Five Freedoms paradigm, and in statements resembling those by FAWC, variously referred to the Freedoms as ideal or aspirational states or principles that provide a logical and comprehensive guide for animal welfare assessment and management (e.g., [9,10,21,22,23]).

It is proposed here, however, that the notion of aspiring to achieve these Freedoms *defined* as *ideal* states and simultaneously viewing them as a *logical and comprehensive guide* for effective animal welfare assessment and management, creates the mistaken expectation among those who are less well informed that such states of freedom are indeed fully achievable. In like manner, non-reflective or formulaic reference to the Five Freedoms, implicitly considered to be fully achievable without impediment by applying the Five Provisions, also occurs in cases were animal welfare receives cursory attention (also see [24]).

The present paper begins by briefly exploring some factors that may have contributed to this misconception. It then considers the following matters: the extent to which the Five Freedoms paradigm captures, in the specifics or the generality of its expression, the breadth and depth of current knowledge of the biological processes that are germane to understanding animal welfare and guiding its management; distinctions between surviving and thriving and the impact of the environment on these states; the importance of differentiating between physical/functional states and unpleasant or pleasant subjective experiences when making animal welfare assessments; the need to have different frameworks for assessing animal welfare compromise and enhancement; the impetus to move towards “a life worth living” understood in terms of a good Quality of Life; the importance of human–animal interactions; and a comparison of key features of the Five Freedoms paradigm, the Five Domains Model for welfare assessment, and Three Orientations identified as different aspects of animal welfare thinking. Finally, the paper concludes by outlining an alternative means of characterising animal welfare and its management.

## 2. The Five Freedoms and the Elimination of Negative Experiences

Four overlapping factors are suggested here to have contributed towards some animal advocate groups and others interpreting the Five Freedoms as being completely achievable. First, there was the engaging alliterative nature of the term itself and its persuasive focus on the evocative notion of “freedom”. Indeed, these attributes led John Webster to retain the term when he extended the paradigm to include additional features of significance to animal welfare [13].

Second, the apparently straightforward character of each Freedom as stated made them easy to grasp [12], and this undoubtedly contributed to their widespread adoption. During at least the last 15–20 years, however, they have usually been presented in an abbreviated format, such as in Table 1, mostly without reference to the caveats that had been clearly stated in 1993/1994. Indeed, since 1994 the Five Freedoms have been published using this restricted format on hundreds of occasions. The Freedoms and aligned Provisions therefore became the primary focus, leading, it is suggested here, to a marked decline in awareness of the constraining caveats, and thereby leaving the way open for notions of “complete freedom” to emerge.

Third, it is apparent that under such circumstances the word “freedom” applied to “freedom from negative experiences or states” could easily progress to represent a complete absence of those experiences or states. Clearly, the adoption of this notion as a valid foundation for good animal welfare management would bring with it the implicit or explicit expectation that it is practically possible for the named negative experiences or states to be eliminated. Moreover, it would follow that animal care staff would have an obligation to keep animals completely free of these experiences or states at all times, and (wrongly) could be censured if they did not do so.

Fourth, animal rights ethical theories achieved greater prominence during the same period (e.g., [25,26]) and might have had some impact in this context. Among other points, the right of animals to be completely free from all harms done to them by people was emphasised (e.g., [27]). Thus, it may be seen that, implicitly or explicitly, the Five Freedoms could easily be assigned the status of “rights” (e.g., [28]) or “fundamental freedoms”, not only reflecting some aspects of such animal rights thinking, but also reflecting a pervasive, yet unconscious, alignment with the same phraseology used in the United Nations Universal Declaration of Human Rights [29].

## 3. The Five Freedoms and Contemporary Biological Understanding

It is obvious biologically that even during short periods of its life an animal is never likely to be completely free of the stipulated negative experiences or states of thirst, hunger, discomfort, pain, fear, distress, malnutrition, disease and injury [13,24]. Human experience and common sense reinforce this conclusion. Nevertheless, a major strength of the Five Freedoms paradigm was that it very effectively directed attention towards the need to understand, identify and minimise negative welfare states, and this aligned with the major focus of most animal welfare science activity during the last two decades [30,31,32]. One outcome was a huge improvement in knowledge of the functionality of animals relevant to their welfare, and this now provides a basis for considering whether or not the concept of the Five Freedoms can capture sufficient up-to-date understanding to be useful. Presented below are several key points that, taken together, help to clarify this matter.

### 3.1. Negative Subjective Experiences of Two Main Types have been Identified

Note that research during the last 15–20 years has expanded the list of potential negative subjective feelings or emotions, generically known as affects or affective states, that most mammals and some birds are now considered likely to experience [33,34,35]. Moreover, two major types of sensory inputs that give rise to these experiences have been clarified [35,36,37]. First, there are negative affects generated mainly by sensory inputs that register imbalances or disruptions in the internal physical/functional state of an animal, and these include breathlessness, thirst, hunger, pain, nausea, dizziness, debility, weakness and sickness. Second, there are other negative affects, which are associated mainly with sensory inputs that contribute to an animal’s cognitive assessment of its external circumstances, and these include anxiety, fear, panic, frustration, anger, helplessness, loneliness, boredom and depression. Behavioural, physiological and neuroscience evidence supports these observations (e.g., [33,38,39,40,41]).

### 3.2. Particular Negative Affects Elicit Behaviours that are Essential for Survival

During the same period it was recognized that negative affects, especially those associated with the internal physical/functional state of animals, are essential components of genetically embedded behavioural mechanisms designed to secure the survival of the animals [36,40,42]. The undoubted negativity of each of these affects creates a sense of urgency to engage in behaviours that are specific to each affect, for example, breathlessness elicits increased respiratory activity, thirst provokes water seeking and drinking, hunger food acquisition, and pain escape or avoidance responses to injury [36,37,43]. Moreover, the greater the intensity of the negative affect, the greater is the sense of urgency to engage in the aligned behaviour, and vice versa, so that once the behaviour achieves the required physical/functional outcome the intensity of the negative affect declines [36].

Animals are therefore genetically preprogrammed to experience these negative affects and without them they could not survive. These observations provide a more nuanced understanding of the mechanisms involved and show more coherently why eliminating these survival-critical negative affects is not possible. Animals under human control therefore need to be managed in practical ways that avoid extremes of such experiences by keeping their intensity within tolerable limits that nevertheless still motivate the essential life-sustaining behaviours [37,44].

### 3.3. Minimising Survival-Critical Negative Affects does not Guarantee an Overall Affective Balance that is Positive

It has been widely assumed that when animals are managed with attention specifically focused on the survival-critical negative affects named in the Five Freedoms paradigm, *i.e.*, thirst, hunger, distress, discomfort and pain, their net affective experience will be positive. However, the situation is not that straightforward. This is because management actions that minimise only the negative affects elicited by imbalances or disruptions of internal physical/functional states can at best merely neutralise each such negative experience [35,36,37]. Thus, with regard to the wider list of survival-critical negative affects outlined above, high intensity breathlessness can at best be neutralised, and then only temporarily, and likewise with regard to thirst, hunger, pain, nausea, dizziness, debility, weakness and sickness. Of course, relief from these negative affects after they have been experienced at high intensity might be perceived as hedonically positive, but this is likely to be short-lived [37,42]. Optimal management of these affects should therefore be aimed at keeping their intensity in the range between low tolerable levels and neutrality. Note moreover that minimising these negative affects appears to have a permissive role in removing impediments to animals engaging in behaviours they may find rewarding (see subsection 3.5).

### 3.4. Environmental Improvements can Replace Other Negative Affects with Positive Ones

As noted above, a second type of sensory input contributes to the brain processing that underlies an animal’s cognitive perception of its external circumstances [35,37]. At the negative extreme, close confinement and isolation of social animals in threatening and/or barren environments may lead to experiences that include various combinations of anxiety, fear, panic, frustration, anger, helplessness, loneliness, boredom and depression [30,33,37,45]. In contrast, keeping social animals with congenial others in spacious, stimulus-rich and safe environments provides them with opportunities to engage in behaviours they may find rewarding. These behaviours include, but are not limited to (see section 4), environment-focused activities of exploration and food acquisition (foraging or hunting), and the animal-to-animal interactive activities of bonding and bond affirmation, maternal, paternal or group care of young, play behaviour and sexual activity [44,46]. In general terms, the associated positive affects are considered likely to include various forms of comfort, pleasure, interest, confidence and a sense of control [30,33,47]. More specifically, they may include feelings of being energised, engaged, affectionately sociable, rewarded maternally, paternally or as a group, nurtured, secure or protected, excitedly joyful and/or sexually gratified [38,40,44,46]. Understanding this should increase the drive to provide animals with environments that are improved in these and other ways, especially as this is likely to achieve longer term and more varied beneficial welfare outcomes. Of course, as with negative affects, the duration, intensity and frequency of animals’ experiences of such positive affects are likely to vary [46].

### 3.5. When the Intensity of Survival-Critical Negative Affects is Significant Animals are less Motivated to Engage in Rewarding Behaviours

The brain processing that underlies the generation survival-critical negative affects and that which underlies the generation of situation-related positive affects interact, such that when animals experience significant levels of the former this discomfort inhibits their motivation to utilise existing opportunities to engage in behaviours that would be rewarding [38,42,48,49]. Examples include the following: significant acute or chronic pain caused by traumatic injury or pathological processes may lead to immobility, restricted movement or otherwise impaired behavioural responsiveness to potentially pleasurable opportunities; breathlessness caused by acute or chronic cardio-respiratory or respiratory impairment may restrict animals to low levels of physical activity, thereby hindering their capacity to, for example, explore actively, hunt vigorously or forage extensively; and sickness, weakness, nausea, dizziness and other debilitating affects may demotivate animals from engaging in physically active and gregarious behaviours, thereby leading them to remain inactive and isolated from others [37]. Thus, therapeutic intervention should minimise such negative affects to both reduce the associated welfare compromise and to encourage the utilisation of existing opportunities for welfare enhancement [37].

### 3.6. Integrating these Points

The following points may now be highlighted. First, the list of 18 negative affects currently considered to be variously relevant to the welfare of mammals and birds far exceeds the six included in the Five Freedoms paradigm (Table 1). It is acknowledged that the word “distress” would capture many of these additional affects. However, its lack of specificity hinders recognition of the need to focus more directly on these affects and thereby to better manage each of the conditions that gives rise to them [34,50,51]. Second, the identification of two major types of such affects, *i.e.*, survival-critical and situation-related negative affects, is an important distinction not apparent within the Five Freedoms paradigm. This distinction is especially important as the former negative affects can, at best, usually only be temporarily neutralised by appropriate interventions, whereas the latter can be replaced by positive affects when animals’ circumstances allow them to engage in behaviours they find rewarding [37,44]. Moreover, these two types of experience interact such that the presence of survival-critical negative affects at significant intensities demotivates animals from engaging in behaviours that they may otherwise find rewarding [37]. Third, opportunities to engage in rewarding behaviours are provided by stimulating environments, which, when appropriately configured, enable animals to experience various forms of comfort, pleasure, interest, confidence and a sense of control [2,30,37]. This point is obviously aligned with Freedom 5, “to express normal behaviours”, and Provision 5, “by providing sufficient space, proper facilities and company of the animal’s own kind”. However, the range of specific provisions required for animals to experience the wide variety of the positive affects that is available to them when they are not restricted by constraining human management approaches (e.g., [2,30,45,46,47,52]) may now be seen to considerably exceed that envisaged in 1993/1994.

Overall, therefore, quite apart from the potential for the Five Freedoms paradigm to be misunderstood or misrepresented as aiming for the complete elimination of negative experiences, it does not capture, either in the specifics or the generality of its expression, the breadth and depth of current knowledge of the biological processes that are germane to understanding animal welfare and to guiding its management.

## 4. Surviving, Thriving and Environments that Enhance Welfare

It is helpful to distinguish between those husbandry practices directed mainly at keeping animals alive and those aimed at achieving more than mere survival, *i.e.*, those that enable animals to thrive [53]. The former practices focus on meeting animals’ basic needs for water, food, shade/shelter and disease reduction. It is now well understood, however, that a minimalist focus on the basics for survival cannot sufficiently reduce the intensity of negative affects in the nutritional, environmental and health domains to sustainably secure net welfare states that are positive [5,13,30,33,34,37,54]. For example, it is possible to be chronically underfed or exposed to meteorological stressors, or to have chronic injuries or infections, and still be alive.

More is required. This can be achieved in two ways: the first is to manage animals in ways that both reduce survival-critical negative affects to tolerably low levels, thereby exceeding the minimum required for basic survival, and the second is to provide improved environments that offer the animals greater opportunities to experience positive affects [44]. These improvements include making available the following opportunities: variable environments with a congenial balance between predictability and unpredictability; access to preferred sites for resting, thermal comfort and voiding excrement; environmental choices that encourage exploratory and food acquisition behaviours which are enjoyable; a variety of feeds having pleasurable tastes and textures; and circumstances that enable social species to engage in bonding and bond affirming activities and, as appropriate, other affiliative interactions such as maternal, paternal or group care of young, play behaviour and sexual activity [2,30,33,37,54]. Overall, the objective is to provide a range of opportunities for animals to experience comfort, pleasure, interest, confidence and a sense of control.

Environments that may provide welfare enhancing opportunities such as these range from being somewhat improved to stimulus rich, and may also include pleasurable interactions with animal care personnel and others (see section 8). Such opportunities are apparent in most animal use sectors, but their extent varies widely both within and between sectors, in part due to constraints imposed by the primary roles of each sector. Three contrasting examples illustrate this.

### 4.1. Zoo and Aquarium Sector

Simply keeping animals alive posed major challenges for most zoos during the many centuries when they mainly functioned as menageries for displaying little known or unusual wild animals [55]. This was also the case from the early-to-mid 19th century when zoo and aquarium animals began to be studied scientifically, and from the mid-20th century when conservation of threatened species was adopted as an increasingly important role [55]. However, concern about the welfare of such captive animals is much more recent, interest in it having burgeoned only during the last 15–20 years. Now, the zoo and aquarium sector is strongly committed to the worldwide promotion of positive welfare states in the animals in its care [56]. Leading zoos in particular have established innovative welfare enhancing programmes, which incorporate what they usually call environmental or behavioural enrichments, and which their staff strongly support and seek to continuously extend and improve (e.g., [53,57,58,59]). The numerous successful enrichments developed by leading zoos provide examples of how the many less advanced zoos could improve their animal welfare management and could thereby help to reduce the wide variability in standards still apparent across the whole sector (e.g., [56]).

### 4.2. Livestock Farming Sector

Between 60 and 80 years ago, numerous nutritional, environmental, disease and other problems were poorly understood and farmers struggled to keep their livestock alive and healthily productive [13,33,34]. This motivated several decades of problem-based research into body mechanisms and their manipulation which led to the development of numerous strategies for more effectively meeting animals’ basic survival needs [34,60]. This success reduced some major negative impacts on the animals, but the related implications for their welfare only became a focus for serious scientific enquiry 30–40 years ago [13,33,34,61]. Initially the livestock sector, perhaps especially intensive farming enterprises, resisted all but quite limited environmental improvement initiatives [62], viewing many of them to be scientifically unsupported, impractical, and potentially costly impositions on commercial enterprises that need to remain financially viable [47]. However, during the last 10–20 years, national (e.g., [15,16,17]) and international (e.g., [5,21,63,64]) regulations or codes of welfare have increasingly included provisions that extend the welfare management focus to include elements well beyond the basic survival needs of farm animals [47]. It is interesting to consider that the farming sector might find it instructive to keep under review the many innovative enrichments zoos develop as these might seed ideas for the implementation of practical, effective and economic farm-based initiatives.

### 4.3. Animal-Based Research Sector

The use of animals for scientific purposes has long emphasised the importance of uniformity with regard to the health status, physiological state, age/maturity, genetic background and other features of the animals, as well as their environment, in order to more effectively demonstrate treatment effects. Yet, the discriminative advantage of this uniformity is increasingly being regarded as hindering extrapolation of study outcomes to populations of genetically and otherwise more diverse animals of the same or different species living in more variable environments [65,66,67,68]. Moreover, the barren, space-restricted containers or enclosures in which the majority of laboratory animals such as rodents and rabbits are still kept are recognised as significant welfare imposts in themselves in addition to those due to some of the manipulations that form the primary focus of each study [65,67,69,70]. However, the impetus to improve laboratory settings, considered to have potential benefits as additional refinements aligned with the Three Rs tenet of replacement, reduction and refinement, is increasing as more information that facilitates their careful introduction becomes available (e.g., [37,50,67,71]).

### 4.4. Incremental Introduction of Environmental Improvements

When considering increasing the opportunities for animals to have rewarding experiences, for example, in zoos and aquariums, on farms and in laboratory settings, it would obviously be unrealistic practically and economically to aim to deliver all such opportunities immediately. However, it does seem reasonable to suggest, in accord with the principle of incremental improvement [72], that those responsible for animal care and management in all animal use sectors should utilise information that is now widely available (e.g., [4,30,37,45,52,54]) to guide the speedy implementation of whatever small, medium or large changes may be feasible in their particular circumstances [44].

## 5. The Five Domains Model, Physical/Functional States and Affective Experiences

The Five Freedoms paradigm did not differentiate between the physical/functional (malnutrition, exposure, disease and injury) and affective (thirst, hunger, discomfort, pain, fear and distress) elements of animal welfare. This, and the orientation towards desired states of freedom, hindered use of the Freedoms as a means to coherently and systematically identify and grade different types of negative welfare impact, because the meaning of the notion of “degrees of impaired freedom” was inherently obscure, and therefore lacked utility.

The Five Domains Model for assessing animal welfare compromise, initially applied to animals used in research, teaching and testing, was developed by Mellor and Reid to address these problems [73]. Furthermore, it was designed to provide a more thorough, systematic and comprehensive means to assess negative welfare impacts.

**Figure 1:**
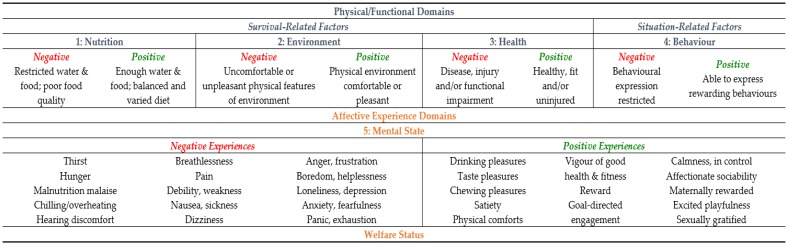
An abbreviated version of the Five Domains Model. It summarises survival-related and situation-related factors and their associated physical/functional domains, and provides examples of negative or positive affects assigned to the mental domain. The overall affective experience in the mental domain equates to the welfare status of the animals. For full details see [37].

Taking a predominantly physiological orientation, the Model was structured to first evaluate particular physical/functional disruptions and imbalances, as well as restrictions on behavioural expression, and then to identify the specific negative affects each disruption, imbalance or restriction would be likely to generate. Accordingly, the Model incorporated four predominantly physical/functional domains of “nutrition”, “environment”, “health” and “behavior”, and a fifth “mental” domain for focusing attention on all of the individual negative affects identified and their accumulated overall impact on welfare. The net affective outcome in the “mental” domain therefore represented the animals’ overall welfare state (Figure 1).

Since its formulation in 1994, the Five Domains Model has been regularly updated to incorporate developments in animal welfare thinking, for example, by the inclusion of additional negative affects to give greater definition to the catchall term “distress”, and to apply the Model to animal uses beyond research, teaching and testing, for example, to the management of farm, companion, sports, service, draught and wild animals [32,34,72,74,75,76,77]. Finally, the most recent Model update (Figure 1) has incorporated comprehensive consideration of a broad range of positive affects and how they are generated (see: [35,37,44]).

As originally formulated the Model appears to have anticipated at least two later developments in animal welfare thinking and assessment. First, the Model integrated elements of the “biological functioning” and “affective state” ways of understanding animal welfare, which at the time were competing schools of thought (subsection 9.3), and it thereby implicitly anticipated the later acceptance that these two elements in fact interact dynamically as part of the integrated operation of animals’ bodies as a whole [33,34,78]. And second, in like manner, the first four physical/functional domains of the Model anticipated the later focus of the European Welfare Quality assessment system on the categories of “good feeding”, “good housing”, “good health” and “appropriate behavior” [79,80]. This was understandable as these four domains or categories were obvious foundational elements of the Five Freedoms. With the Welfare Quality system, however, the affective outcomes to be minimised or promoted were not categorised separately; rather they were included among a list of 12 explanatory criteria aligned with these four named categories [79,80].

## 6. Different Frameworks are Needed to Assess Welfare Compromise and Enhancement

### 6.1. Welfare Compromise

The defining point of reference for animal welfare compromise is the most intensely unpleasant affects animals may experience, equated with severe suffering [34], and the primary objective of assessing compromise is to facilitate the application of management practices designed to minimise those experiences [37]. Of course, it is first necessary to identify particular welfare problems and their related measurable indices, and then to devise remedies, activities which have preoccupied animal welfare and other scientists for several decades (e.g., [13,33,60,61,78,81]).

Most indices of compromise include behavioural, anatomical, physiological, pathological and clinical diagnostic parameters (e.g., [82,83,84]). Such indices, which are aligned with the survival-related factors within the nutritional, environmental and health domains of welfare, mainly provide information about negative-to-neutral states, *i.e.*, the presence or absence of physical/functional disruptions and, if present, their severity [34]. Usually, therefore, these indices are not informative about the likely affective constituents of positive welfare states [41,44]. This highlights a significant disjunction, also recognised by others [41,54,85], and this needs to be acknowledged.

Note also that some of the indices of physical/functional disruptions, for example, circulating stress hormone levels [86], can be useful for assessing animals’ negative cognitive perceptions of their external circumstances, with the proviso that confounding factors that may otherwise elevate such levels can be excluded. If so, as with assessing affective impacts of internal disruptions, their use would apply to affects with valences in the negative-to-neutral range, not positive affects. This is because stress hormone concentrations and related parameters cannot decrease below stress-free baseline levels [86], and this constrains their interpretative scope when used to assess the potential benefits of environmental improvements [86,87]. Thus, a decline in stress hormone levels towards baseline values may simply be because the improvements introduced have made what was affectively very unpleasant close confinement in barren conditions somewhat less unpleasant, or, at best, tolerably neutral. Furthermore, the persistence of baseline stress hormone levels in the presence of such improvements does not indicate that they are of no benefit. At most it would suggest that the cognitive awareness of the animals was not dominated by one or more stress-inducing negative affects.

### 6.2. Welfare Enhancement

Adopting a new defining point of reference for welfare enhancement has therefore been recommended [37,44]. Its focus is on “positive affective engagement” [44], not the absence of suffering [34], as the absence of suffering does not necessarily deliver good welfare states [44]. The concept of “positive affective engagement” is intended to capture the experience animals may have when they actively respond to motivations to undertake behaviours they find rewarding, and it potentially incorporates all of the associated affects that are positive [44,46]. Thus, it includes the genetically pre-programmed, or learned, affectively positive impulses to engage in rewarding behaviours, and it also includes positive affects related to anticipation, goal achievement and memory of success [40,41,42,85]. It is argued that these experiences provide sufficient justification in and of themselves to frame a reference standard that acknowledges the importance to animals of having opportunities to express these behaviours [46,47].

Importantly, “positive affective engagement” aligns with the rewards animals may experience when exercising “agency”. Agency is apparent when animals engage in voluntary, self-generated and goal-directed behaviours [88] and is linked to a general sense of being in control [89]. Thus, “positive affective engagement” includes the rewarding content of an animal’s experiences whilst exercising agency. Note, however, that positive experiences may also arise in ways not directly related to the exercise of agency [37].

It is apparent, therefore, that the most informative indices of animals having positive affective experiences are behavioural [44,46] and that these may be identified when animals utilise stimulating opportunities to engage in activities they find rewarding [47]. As noted above, such behaviours include, but are not limited to (section 4), some features of the environment-focused activities of exploration and food acquisition (foraging or hunting), and some elements of animal-to-animal interactive activities related to bonding and bond affirmation, maternal, paternal or group care of young, play behaviour and sexual activity [44,46].

## 7. Towards a “Life Worth Living”

The concept of Quality of Life (QoL), considered in detail elsewhere (e.g., [32,90,91]), highlights that animals have both positive and negative experiences and it focuses on the balance between the two. Notwithstanding significant difficulties in making precise QoL assessments [32], preferred states are recognised as those where, overall, positive experiences would predominate. In line with this, and as a means of augmenting the drive to promote positive welfare states, the UK Farm Animal Welfare Council developed the notions of “a life not worth living”, “a life worth living” and “a good life [2]. Building on this, and related additional ideas [92], Green and Mellor [32] formulated a four-tier QoL scaling system with two positive categories above and two negative categories below a neutral point of balance (Table 2). As they also considered the utility of this scale in some detail only brief comment will be made here, except to note that, at present, it seems more likely to be effective as a motivational framework than as an effective foundation for developing regulations [32].

It is apparent that the highest welfare status within the range represented by the category of “a life worth avoiding” would be one that closely approaches the “neutral point of balance” from below. In light of observations made here, this may be the best outcome achievable when there is full compliance with those codes of welfare or practice that, in the past, were directed almost entirely at the survival-critical features of welfare management related to the nutrition, environment and health domains, but not behaviour. Examples include some of the early codes for caged layer hens and pregnant sows which, among other limitations, accepted very severe restrictions on their movement. Thus, in order to improve on merely neutralising negative survival-critical affects, and to contribute to the drive to secure “lives worth living”, codes would obviously need to include minimum standards specifically aimed at promoting positive experiences. Examples of such codes already exist [8,15,16,17,21,63,64], but further improvements are likely to be possible [30,47,54,93]. It is recommended that all future reviews of codes, no matter how advanced they currently appear to be, should, on a species-specific basis, include explicit and detailed consideration of whether or not the current minimum standards need to be extended to ensure that they do indeed enable animals to have “lives worth living”.

Such reviews would clearly entail considerable discussion and would need to include consideration of what constitutes “a life with living”. Those discussions could be facilitated by referring to extensive lists of potential positive affects, and their generation, for example, those included in the latest version of the Five Domains Model for animal welfare assessment [37]. Allied to this, there is also a list of questions that was prepared to aid this process [2,47]. Overall, what opportunities have been provided for the animals’ comfort, pleasure, interest and confidence? More specifically, what provisions have been made to ensure that consuming the food provided will be an enjoyable experience? How will expressions of normal behaviour be encouraged and harmless wants met? What environmental choices will be available that will encourage exploratory and food acquisition activities which are rewarding? And what provisions have been made to enable social species to engage in bonding and bond affirming activities and, as appropriate, other affiliative interactions such as maternal, paternal and group care of young, play behaviour and sexual activity?

Finally, the notion of QoL acknowledges that animals can have both negative and positive experiences, and that the net balance between them will vary over time [32]. Occasionally welfare challenges will exceed the capacity of routine husbandry and clinical management to maintain negative affects at tolerably low levels, thereby requiring remedial interventions. Also, some such interventions may themselves elicit negative experiences (e.g., some transient post-surgical pain), but these may be justified by longer-term welfare improvements (e.g., restoration of pain-free mobility after a bone fracture heals) [2]. Thus, even with the best animal management, the character and intensity of negative experiences would be expected to vary over time. So also would the character and intensity of positive experiences [46]. It follows that, just as the aim of completely eliminating negative affective experiences is acknowledged to be unrealistic, it is equally unrealistic to expect that the promotion of positive affective engagement could result in continuously pleasurable experiences. Rather, both will vary, so the objective should be to achieve a net balance over time that favours positive experiences [2,32,46,47].

## 8. Human–Animal Interactions may Enhance Welfare

Humans have almost complete control over food and water availability, quality and variety, as well as other important features such as the space, environmental complexity and social groupings provided for many domestic and captive wildlife species (e.g., [56,94]). The decisions and behaviour of humans therefore have the potential to both compromise and enhance animal welfare. Indeed, it has been argued that animal care personnel can have the most influence on an animal’s welfare status [95]. This is because they must be able to anticipate problems, identify when problems have occurred and apply remedies, and also be able to identify, maintain and/or promote good welfare. Clearly, the person’s knowledge, attitudes, skills, training and familiarity with the animals are important, but so are broader issues such as job motivation and satisfaction, working conditions, actions of co-workers and organisational policies and rules [95,96]. Importantly, targeted cognitive-behavioural training can improve attitudes and behaviour towards animals, with consequent improvements in animal handling, welfare and, in the case of livestock, productivity [95]. The promotions of “lives worth living” among animals in human care and control must therefore include consideration of these key features of human influence. Finally, an additional related factor which often has significant welfare benefits, especially with “hand-on” management of small numbers of animals such as occurs in zoos, in the home and as part of recreational sporting activities, is the development of a close human–animal bond [46]

## 9. The Five Freedoms, Five Domains and Three Orientations

It is helpful to compare key attributes of the Five Freedoms paradigm, the Five Domains Model and the Three Orientations towards animal welfare thinking in order to consider their various contributions to the development of scientific understanding in this arena.

### 9.1. The Five Freedoms

The Five Freedoms may be viewed as an early paradigm that facilitated thorough consideration of animal welfare and its management. Its wide adoption during the last two decades may be attributed to four key factors, namely that it: (1) scoped the wider dimensions of animal welfare, including subjective experiences, health status and behaviour; (2) specified areas of welfare concern in terms of particular negative experiences (thirst, hunger, fear, distress, discomfort, pain) and states (malnutrition, injury, disease, behavioural expression); (3) defined five particular targets for welfare improvement—the Freedoms; and (4) provided practical advice on how these targets might be achieved—the Provisions.

When formulated, the paradigm was motivational, informative, easily understood and practically useful, hence its wide adoption. It was recognised at the time, however, that the focus on “freedom”, even understood as animals being “as free as possible from” particular welfare problems, did not provide a cogent basis for grading the severity of such problems (Mellor and Reid 1994), because the meaning of the notion of “degrees of impaired freedom” was inherently obscure, and therefore lacked utility. Moreover, not distinguishing between negative experiences (affects) and the internal states or external circumstances that give rise to them was a further hindrance to the Freedoms providing a systematic and biologically coherent way to grade the severity of welfare problems [73].

### 9.2. The Five Domains Model

The Five Domains Model (section 5) was developed by Mellor and Reid [73] to address these difficulties by providing a means to comprehensively, systematically and coherently identify and grade the severity of different forms of welfare compromise, understood in terms of the internal functional and external situational origins of negative affective experiences. During the last two decades the Model has been updated and extended as scientific knowledge of animal welfare has become more detailed and nuanced (e.g., [34]). The most recent update incorporates ways that facilitate the grading of *both* welfare compromise (related to negative experiences) and welfare enhancement (related to positive experiences) [37]. Thus, the Model is a facilitatory grading device. It is based on current scientific understanding of animal welfare and provides numerous examples of welfare compromise and enhancement. It is not a definition of animal welfare. Indeed, for reasons noted below (section 10), it is considered preferable to *characterise* animal welfare, rather than attempt to *define* it [34].

### 9.3. The Three Orientations

Three different orientations have been identified as influencing the focus of science-based thinking about the nature of animal welfare and its management [33,97,98]. They were biological function, affective state and natural living. The biological function orientation was dominant initially. It emphasised well-understood physical/functional states and physiological mechanisms, but not subjective experiences or affects. The focus was on assessing hindrances to achieving biological fitness, resilience and performance. Subsequently, the affective state orientation increased in influence as the subjective experiences of animals became more widely accepted as a legitimate area for scientific investigation. Particular attention was given to identifying and interpreting animals’ motivations, preferences, aversions and aligned behaviours. Finally, overlying the biological function and affective state orientations throughout has been the natural living orientation. It draws attention to the anticipated good experiences animals may have in circumstances that exclude human-imposed environmental restrictions.

Until about 10 years ago these three orientations appeared to be competing frameworks that gave rise to apparently conflicting conclusions. Now, however, the first two, biological function and affective state, are widely accepted to be dynamically integrated elements within the body operating as a whole entity [32,78]. For example, it is understood that animal welfare relates to the affective experiences animals may have and that these reflect the operation of biological mechanism linked to physical/functional states within the body and to other mechanisms underlying the brain processing that gives rise to animals’ cognitive perception of their external circumstances. Integrated activities at the biological function/affective state interface are therefore now widely accepted as being the key to understanding the manifestation of animal welfare compromise and enhancement and ways for assessing them. Finally, the natural living orientation remains a reference point by which likely untoward consequences of imposed environmental and other restrictions can be identified and assessed using frameworks that incorporate the two other orientations as integrated elements [32,47], for example, the Five Domains Model [37].

## 10. An Updated Characterisation of Animal Welfare

During the 30 years since animal welfare emerged as a legitimate area of scientific study no universally endorsed definition of it has emerged. Expressed in general terms, different definitions have emphasised coping with challenges from natural and unnatural environments, fitness and health, meeting biological needs and wants, and recognition of the significance of subjective sensory or emotional experiences, feelings or affective states [12,98,99,100,101]. As illustrated here, and noted elsewhere [32], key interacting reasons for this diversity include improvements in the understanding of animal functionality over time, the introduction of fresh perspectives by researchers specialising in different disciplines, and changes in dominant societal views on what constitutes acceptable and unacceptable ways of treating animals.

Ideas evolve in animal welfare science, as in all other disciplines, so that current definitions will inevitably need to be revised or replaced at some later date. A preferable approach may be to *characterise* animal welfare in terms of its major features that are accepted at each time [34]. This avoids the potential inflexibility and defensiveness that definitions sometimes attract, allows well-accepted notions to be included and, as ideas change, for related features of the characterisation to be revised appropriately or discarded. Presented here for consideration and critique is an updated characterisation of the principal features of animal welfare based on considerations enunciated above and elsewhere [32,34,35,44,78].

### 10.1. General Features

- Animal welfare is a state that is subjectively experienced by an animal; it is a state within the animal.
- Animals of welfare concern have brains or equivalent neural structures that are sufficiently sophisticated to support, as a minimum, conscious perception of and behavioural responses to a range of sensory inputs.
- Only species with sufficiently evolved neural structures (phylogenetic stage), and, within those species, only individuals where sufficient neural development has occurred (ontogenetic stage), are therefore of welfare concern.

### 10.2. Generation of Subjective Experiences

- As animal welfare relates to experienced sensations or perceptions, the animal must be conscious; unconscious animals cannot experience anything.
- Conscious experiences of welfare interest are negative or positive.
- These experiences arise as the integrated outcomes of sensory and other neural inputs from within the animal’s body and from its environment.
- These inputs are processed and interpreted by the animal’s brain according to its species-specific and individual nature, and past experience.
- The integrated subjective outcome represents the animal’s current experience (*i.e.*, its welfare status), and this changes as the balance and character of the inputs change.
- An animal’s welfare status at any one time may vary on a continuum from very bad to very good.

### 10.3. Specific Subjective Experiences (Affects or Affective States)

- There are two major types of negative affective experiences: those that reflect imbalances or disturbances in the internal physical/functional state of the body, and those elicited from outside the body that contribute to an animal’s perception of its external circumstances.
- Internally generated negative affects include breathlessness, thirst, hunger, pain, nausea, dizziness, debility, weakness and sickness. Each of these affects motivates animals to behave in particular ways that help to secure their survival. However, correction of the associated imbalance or disturbance, whether achieved by the animal unaided or with support from animal care staff, at best will usually only result in a neutral, not positive, affective outcome.
- Externally generated negative affects include anxiety, fear, panic, frustration, anger, helplessness, loneliness, boredom and depression. These are mainly elicited by threatening, cramped, barren and/or isolated circumstances, and will persist for as long as such conditions prevail. These are situation-related negative affects and human intervention is usually required to correct them.
- Providing animals with opportunities to engage in behaviours they find rewarding can replace situation-related negative affects with positive experiences. Such opportunities become available when social animals are kept with congenial others in spacious, stimulus-rich and safe environments.
- Rewarding behaviours may arise when the key attributes of animals’ environments include, but are not limited to, the following: variability that provides a congenial balance between predictability and unpredictability; access to preferred sites for resting, thermal comfort and voiding excrement; environmental choices that encourage exploratory and food acquisition behaviours which are enjoyable; availability of a variety of feeds having pleasurable tastes and textures; and circumstances that enable social species to engage in bonding and bond affirming activities and, as appropriate, other affiliative interactions such as maternal, paternal or group care of young, play behaviour and sexual activity. Expressed in general terms, the associated positive affects are likely to include various forms of comfort, pleasure, interest, confidence and a sense of control.

### 10.4. Animal Care: Management of Animal Welfare

- All affective experiences of welfare interest are subjective and therefore cannot be measured directly, but there are numerous indirect indices of such experiences that are informative.
- Validated indices of negative welfare states detect anatomical, physiological, pathological, clinical, behavioural and other responses to specific welfare challenges. They have been critically evaluated as useful for guiding preventative and remedial actions in many animal management contexts.
- Some behaviour-based measurements of positive welfare states are also well validated and in current use, and science based support is being sought for others. Positive welfare states may be promoted by improving the environment and by taking other initiatives.
- It is therefore not necessary to measure affective experiences directly to be able to manage them practically in ways designed to minimise negative affects and to provide opportunities to have positive ones.
- The duration, intensity and frequency of negative and positive experiences vary. The net balance between negative and positive experiences reflects an animal’s quality of life such that a net positive balance represents a good quality of life, and vice versa. The quality of life of an animal is anticipated to vary both within and between different stages of its life cycle.

### 10.5. Human–Animal Relationships

- Human–animal relationships can have marked effects on animal welfare. Good welfare-related knowledge, skills, attitudes and behaviour towards animals, including bonding with them, can enhance their welfare, fitness and biological performance and help to ensure that animals have lives that are worth living, *i.e.*, lives where they can and do avail themselves of opportunities to have positive experiences.

## Figures and Tables

**Table 1:** The Five Freedoms and Five Provisions for promoting farm animal welfare [12,13,14].

Freedoms	Provisions
1. Freedom from thirst, hunger and malnutrition	By providing ready access to fresh water and a diet to maintain full health and vigour
2. Freedom from discomfort and exposure	By providing an appropriate environment including shelter and a comfortable resting area
3. Freedom from pain, injury, and disease	By prevention or rapid diagnosis and treatment
4. Freedom from fear and distress	By ensuring conditions and treatment which avoid mental suffering
5. Freedom to express normal behaviour	By providing sufficient space, proper facilities and company of the animal’s own kind

**Table 2:** A Quality of Life (QoL) scale where the different categories are defined in terms of the relative balance of positive and negative experiences animals may have (adapted from [32]).

Category	Description
A good life	The balance of salient positive and negative experiences is strongly positive. Achieved by full compliance with best practice advice well above the minimum requirements of codes of practice or welfare
A life worth living	The balance of salient positive and negative experiences is favourable, but less so. Achieved by full compliance with the minimum requirements of code of practice or welfare that include elements which promote some positive experiences
Point of balance	The neutral point where salient positive and negative experiences are equally balanced
A life worth avoiding	The balance of salient positive and negative experiences is unfavourable, but can be remedied rapidly by veterinary treatment or a change in husbandry practices
A life not worth living	The balance of salient positive and negative experiences is strongly negative and cannot be remedied rapidly so that euthanasia is the only humane alternative
